# The New Departure and the Ideal “Filling”

**Published:** 1879-05

**Authors:** W. Mitchell

**Affiliations:** Delaware, O.


### THE "NEW DEPARTURE," AND "THE IDEAL FILLING."

BY W. MITCHELL, D. D. S., DELAWARE, OHIO.

In the February number of the *Dental Cosmos*, there appeared under the above title reversed, an eulogium on "The New Departure," and a very visionary profile of the "Ideal Filling." In regard to the first, the only "embarassment" it is likely to cause, is to the younger members of the profession, who, with all due respect to heads that have silvered in the service and study of our cherished science, are looking to them for something substantial, instead of that that is likely to prove a delusion and a snare. There is where the embarrassment makes itself felt. That it will produce a "revolution," there is no doubt, (about the same kind that our Mexican neighbors indulge in so frequently). Of course there are those, ever ready to try every new hobby, presented by whomsoever it may be, but even they find it best to settle down at last, to that, what has thus far proved to be the sheet anchor of Dentistry. That the "unbiased progressionist will let it go on," we admit, but must add, he having had anything but a happy experience, as the result of operations performed under the rules of the "New Departure," is only too willing to "let it go on," and partakes as sparingly as possible of it, as it passes by.

While many of us may have opinions as to what should constitute a perfect, or "ideal filling," a little reflection will show us how well nigh hopeless are our wishes that we will ever find that substance, that will meet every demand. In the article referred to, the author states "it must be a cement," and complains because gold needs mechanical insertion. I would ask, does not anything in the way of a filling that is placed in a carious tooth, need mechanical insertion? While an almost tight joint between tooth structure and filling may be possible with cement, we can't ignore the fact, that whatever the solvent may be, its evaporation will cause a shrinkage of the filling, or if heat be applied in its manipulation, condensation will inevitably occur upon cooling. That the number of decayed teeth are rapidly increasing is a fact that should incite us to our best efforts, and to combine careful, manipulative ability with those materials that prove most efficacious in replacing structures that have been the victims of morbific action, and "soft, spongy, or brittle enamel edges," when encountered, had better receive the thorough and judicious attention of a sharp chisel or excavator, and be replaced by something a little more substantial.

A material "both tougher and harder than gold" is demanded. That gold lacks the necessary toughness, sounds rather absurd, to say the least; the gold that a tooth is filled with, is the toughest portion of that tooth, take bulk for bulk of tooth structure and gold. I care not in what direction you select your specimen; and submit your gold, and dentine to the strain test, and note the result. That gold will be superceded as a filling for teeth will not be admitted, with our present knowledge of the requirements of a filling material. The old adage, "Prove all things, and hold fast to that which is good," has no better point of application than here; we are aware of what the results have been; what they are; and I think the future will come with its testimony in favor of that noble metal, that has asserted its superiority over all others, for purity and indestructibility, and that it will hold its place pre-eminent above all else.

It must not be inferred that in mentioning gold all other materials for fillings possess no merit. Amalgam, gutta percha and the oxychlorides fill admirably, according to location, cavities, that if left alone, would soon necessitate removal, and restores to years of usefulness, what would otherwise be lost. "Texture, and color of the natural tooth," are appealed for. We will merely pass the first by as the result of a disordered imagination. Knowing how utterly impossible it would be to simulate tooth tissues by artificial means, while in color we might improve on many or all of our filling materials now in use, but the difficulty of producing shades that would harmonize perfectly, when placed in conjunction with living tissue, without causing the one or the other to deviate from the normal, would be a barrier, that with all our knowledge of physical and vital forces, we could hardly hope to surmount.

"Gloss and translucency," are other desideratums, while the productions of a gloss might be possible, not probable. Translucency would be a problem too knotty for the most enthusiastic to grapple with, with much hope for reward. We know too well the process of vitrification necessary to produce the translucency of our best grades of artificial teeth, and art has taught us that nothing but the intense heat of the furnace will bring those, otherwise refractory substances, feldspar, silex, and others, to a state of translucency, and experience has proven that those are the only materials that will receive and retain that gloss of translucency. "Rapidity of manipulation," is also sought after, and while it would be a boon appreciated equally both by patient and operator, can a person be so derelict of natural laws, and scientific demonstrations not to see, that anything that is to stand the test of time, to say nothing of agents, whose destructibility is not surpassed by even time itself; must of necessity be prepared and carried on to completion in a manner that is consistent with the natural order of things. "Cheapness" is loudly clamored for; and in these days of necessarily curtailed expenses, is a very laudable demand, but in answer to this demand, I would respectfully refer the petitioner to Wedgewood's, short, but very able dissertation on cheapness in connection with work of art.

"Perfect harmony with dentine" is also an essential element, and that can only be found in living tissue, for we are all well aware with what facility the teeth receive impressions, and through their nerves and periosteum; convey those impressions to the brain. Heat, cold, sound, and electricity, are each felt and their peculiar sensations are readily realized by those delicate organs, the teeth.

In conclusion I would say, that in the desire for an "ideal filling," natural laws have been ignored, and flghts of fancy have been indulged in, that are hardly admissible in one desirous of holding a foremost position in the ranks of a profes sion that is noted for its regard and appreciation of natural laws, and scientific facts.

